# High Genetic Diversity of Measles Virus, World Health Organization European Region, 2005–2006

**DOI:** 10.3201/eid1401.070778

**Published:** 2008-01

**Authors:** Jacques R. Kremer, Kevin E. Brown, Li Jin, Sabine Santibanez, Sergey V. Shulga, Yair Aboudy, Irina V. Demchyshyna, Sultana Djemileva, Juan E. Echevarria, David F. Featherstone, Mirsada Hukic, Kari Johansen, Bogumila Litwinska, Elena Lopareva, Emilia Lupulescu, Andreas Mentis, Zefira Mihneva, Maria M. Mosquera, Mark Muscat, M.A. Naumova, Jasminka Nedeljkovic, Ljubov S. Nekrasova, Fabio Magurano, Claudia Fortuna, Helena Rebelo de Andrade, Jean-Luc Richard, Alma Robo, Paul A. Rota, Elena O. Samoilovich, Inna Sarv, Galina V. Semeiko, Nazim Shugayev, Elmira S. Utegenova, Rob van Binnendijk, Lasse Vinner, Diane Waku-Kouomou, T. Fabian Wild, David W.G. Brown, Annette Mankertz, Claude P. Muller, Mick N. Mulders

**Affiliations:** * World Health Organization (WHO) Regional Reference Laboratory for Measles and Rubella, Luxembourg, Luxembourg; † WHO Global Reference Laboratory for Measles and Rubella, London, United Kingdom; ‡ WHO Regional Reference Laboratory for Measles and Rubella, Berlin, Germany; § WHO Regional Reference Laboratory for Measles and Rubella, Moscow, Russian Federation; ¶ Israel Ministry of Health, Tel Hashomer, Israel; # Ministry of Health, Kyiv, Ukraine; ** Ministry of Public Health of the Republic of Uzbekistan, Tashkent, Uzbekistan; †† Instituto de Salud Carlos III, Majadahonda, Spain; ‡‡ WHO, Geneva, Switzerland; §§ University of Sarajevo, Sarajevo, Bosnia and Herzegovina; ¶¶ Swedish Institute for Infectious Disease Control, Solna, Sweden; ## State Institute of Hygiene, Warsaw, Poland; *** Centers for Disease Control and Prevention, Atlanta, Georgia, USA; ††† National Institute for Research and Development in Microbiology and Immunology “Cantacuzino,” Bucharest, Romania; ‡‡‡ Institut Pasteur Hellenique, Athens, Greece; §§§ National Centre of Infectious and Parasitic Diseases, Sofia, Bulgaria; ¶¶¶ Statens Serum Institute, Copenhagen, Denmark; ### Institute of Virology, Vaccine and Sera Torlak, Belgrade, Serbia; **** Istituto Superiore di Sanità, Rome, Italy; †††† Instituto Nacional de Saúde Dr. Ricardo Jorge, Lisboa, Portugal; ‡‡‡‡ Swiss Federal Office of Public Health, Berne, Switzerland; §§§§ Institute of Public Health, Tirana, Albania; ¶¶¶¶ Ministry of Health, Minsk, Belarus; #### Health Protection Inspectorate, Tallinn, Estonia; ***** Ministry of Health, Baku, Azerbaijan; ††††† Ministry of Health, Almaty, Republic of Kazakhstan; ‡‡‡‡‡ Rijksinstituut voor Volksgezondheid en Milieu, Bilthoven, the Netherlands; §§§§§ INSERM U404, Lyon, France; ¶¶¶¶¶ WHO Regional Office for Europe, Copenhagen, Denmark

**Keywords:** measles virus, molecular epidemiology, WHO European Region, years 2005-2006, research

## Abstract

During 2005–2006, nine measles virus (MV) genotypes were identified throughout the World Health Organization European Region. All major epidemics were associated with genotypes D4, D6, and B3. Other genotypes (B2, D5, D8, D9, G2, and H1) were only found in limited numbers of cases after importation from other continents. The genetic diversity of endemic D6 strains was low; genotypes C2 and D7, circulating in Europe until recent years, were no longer identified. The transmission chains of several indigenous MV strains may thus have been interrupted by enhanced vaccination. However, multiple importations from Africa and Asia and virus introduction into highly mobile and unvaccinated communities caused a massive spread of D4 and B3 strains throughout much of the region. Thus, despite the reduction of endemic MV circulation, importation of MV from other continents caused prolonged circulation and large outbreaks after their introduction into unvaccinated and highly mobile communities.

## Abstract 2

Importation of viruses from other continents caused prolonged circulation and large outbreaks in the WHO European Region.

The World Health Organization (WHO) has a goal of eliminating measles in the WHO European Region by 2010. The region extends from the Atlantic to the Pacific, including all western and eastern European countries and the former Soviet Republics (Appendix Table). After the separation of Serbia and Montenegro in 2006, the number of countries in the WHO European Region increased from 52 to 53. Within a well-performing case-based nationwide surveillance system, countries with a goal of elimination are expected to reach <1 confirmed measles case per million population per year. To reach this goal countries are expected to achieve measles vaccination coverage of at least 95% with the first dose and at least 80% with the second (*1*). From 1995 through 2005, the number of countries in the WHO European Region that reported >95% coverage with a first dose of measles-containing vaccine increased from 18 (35.3%) of 51 to 31 (59.6%) of 52. In 2005 and 2006, however, at least 40% and 55%, respectively, of the member states had a measles incidence that was above the elimination threshold (Appendix Table). A total of 36,426 and 55,578 measles cases, including 14 and 9 fatal cases, were reported in 2005 and 2006, respectively (Appendix Table). Thus, measles continues to affect a large number of persons, despite enhanced vaccination strategies.

The pattern of measles virus (MV) genotypes, in combination with epidemiologic investigation, contributes to understanding measles transmission and helps distinguish between continuous circulation and importation and limited transmission of the viruses in a certain region (*2*,*3*). Genotype C2 has been continuously detected in the European Region since the early 1970s and is therefore considered to be the indigenous genotype of Europe (*4*,*5*). D6 viruses have been regularly reported from different countries of the European Region since the early 1990s (*4*–*12*), and genotype D6–associated outbreaks or sporadic cases on other continents were mostly due to MV importations from Europe (*13*–*15*) (Figure 1). These observations thus provide overwhelming evidence for the endemic circulation of genotype D6 in Europe at least during the past 15 years. Measles cases in the European Region associated with other genotypes were mostly due to virus importation from other continents

**Figure 1:**
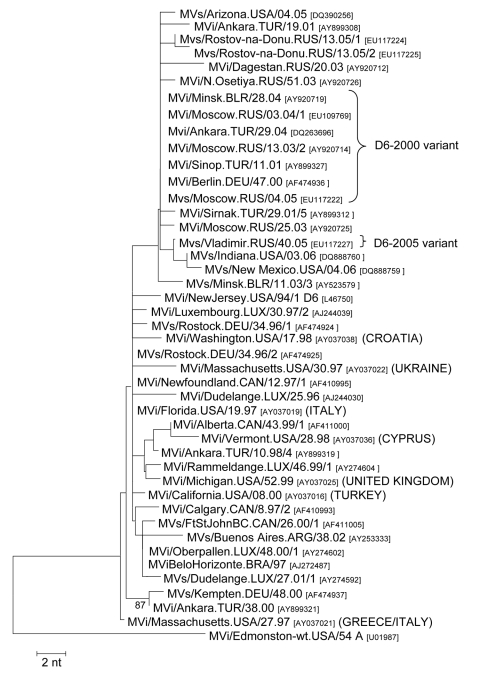
Phylogenetic tree showing representative genotype D6 strains identified in Europe before 2005, strains exported from Europe to other continents (source of exportation is shown in brackets), and the D6 variants that were dominant in Europe during 2005 (D6–2000) and 2006 (D6–2005). The phylogenetic tree was calculated on the basis of the 450 nt that code for the C-terminus of the MV N protein, by using MEGA 3.1 software and the neighbor-joining method (500 bootstraps). Genetic distances are represented as numbers of nucleotide differences between strains. Measles virus strains were named according to World Health Organization nomenclature: MVi/City of isolation.Country/epidemiologic week.year of isolation(/isolate number). Sequences obtained from RNA extracted from isolates (MVi) or clinical material (MVs) were distinguished. GenBank accession numbers are also shown for each strain.

## Methods

Laboratory case confirmation and MV genotyping have increased significantly since the establishment of a global laboratory network for measles and rubella in 2000 (*16*). During 2005–2006, MV genotypes were identified in 25 of the 53 member states of the WHO European Region (Appendix Table). Most epidemics and sustained transmissions were associated with genotypes D6, D4, and B3 viruses (Appendix Figure 1). All strains were genotyped by sequencing the 450 nt that code for the C-terminus of the MV N protein (hypervariable region [HVR]), as recommended by WHO (*17*). Sequencing was performed in different laboratories of the WHO laboratory network for measles and rubella.

## Results

### Genotype D6

**Figure 2:**
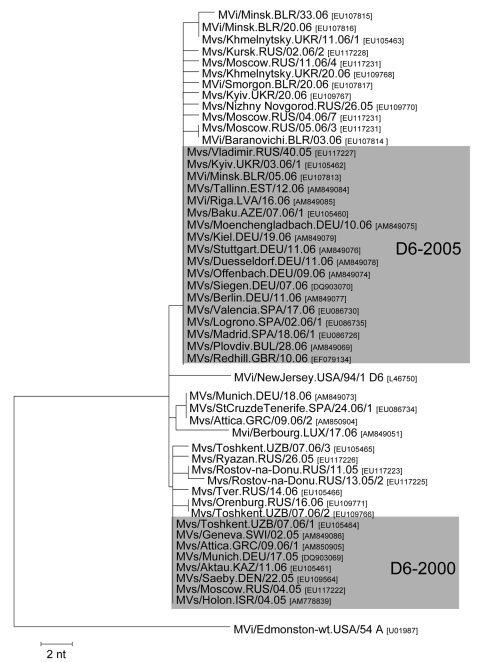
Two main variants, D6–2000 and D6–2005, of D6 identified in Europe during 2005–2006. Tree calculation and measles virus nomenclature are as delineated in Figure 1.

During 2005–2006, genotype D6 viruses were reported from 17 of the 53 countries in the WHO European Region. The overall diversity between these viruses was relatively low (Figure 2), with a maximum genetic distance of 7 nt (1.6%) in the HVR of the N gene. Two main variants, D6–2000 and D6–2005, differing by a single point mutation, accounted for most of genotype D6–associated cases and outbreaks.

The D6–2000 variant was predominant in the Russian Federation throughout 2005 and early 2006 (Figure 2) and was also detected in neighboring Kazakhstan (2006) and Uzbekistan (2006). Moreover, the D6–2000 strain caused outbreaks in Germany (March 2005–July 2005) (*18*) and Greece (September 2005–May 2006) (*19*) and was found in sporadic cases in Denmark (2005), Israel (2005, after importation from Georgia), and Switzerland (2006) (Figure 2). Because the same variant was reported throughout 2000–2005 from different European countries (Figure 1), the cases in 2005 and 2006 were most probably due to endemic transmission of MV in the European Region.

The D6–2005 variant caused the large outbreak in the Ukraine, which had >45,000 cases reported between the last quarter of 2005 and October 2006 (*20*) and was first identified during the last quarter of 2005 in the Russian Federation (MVs/Vladimir.RUS/40.05, Figure 2). The same virus was also found in Azerbaijan during the first quarter of 2006. Of the 32 viruses collected during the Ukraine outbreak, all but 6 were identical to the D6–2005 strain. The other 6 viruses correspond to 3 other variants that differ from the main variant by only single mutations.

Several other measles outbreaks and sporadic cases in 2006 were epidemiologically linked to the Ukraine. Multiple importations of MV from the Ukraine caused different small outbreaks and sporadic cases in Belarus from January through September 2006 (*21*). Among the 47 Belarusian viruses that were sequenced, 37 were identical to the main variant from the Ukraine. The other 10 isolates differed by no more than a single nucleotide from D6 viruses from the Ukraine or the Russian Federation collected during the same period (Figure 2). In Russia in 2006, the main variant from the Ukraine had largely replaced the earlier D6 strains. More than 94% of all genotype D6 strains (n = 88) obtained in the Russian Federation from the last quarter of 2005 and all of 2006 were D6–2005 variants. Although the D6–2005 variant was first reported from the Russian Federation, it may also have been imported from other countries that did not report MV genotypes despite significant numbers of measles cases (Appendix Table).

The D6–2005 virus was also retrieved from a small outbreak in Tallinn (Estonia) during March 2006; the index patient was infected in the Ukraine (MVs/Tallinn.EST/12.06). Similarly, 2 persons infected with the same strain traveled from the Ukraine to Latvia and Bulgaria in April and July 2006, but the virus did not spread. In Spain, the same variant was also obtained from 2 sporadic cases, (MVs/Madrid.SPA/18.06/1 and MVs/Valencia.SPA/17.06), which were epidemiologically linked to the Ukraine, as well as from a small outbreak occurring in La Rioja (MVs/Logrono.SPA/02.06/1) from December 2005 through January 2006 (*22*).

In Germany, the D6–2005 variant caused 2 sporadic cases in Kiel and Stuttgart as well as a major outbreak in North Rhine Westphalia (MVs/Moenchengladbach.DEU/10.06 (*23*,*24*) and a small outbreak in Berlin. Although the index cases of the 2 outbreaks were not found, sequence identity with the main Ukrainian variant suggests that they were directly or indirectly linked to the outbreak in the Ukraine. In the United Kingdom (UK, March–July 2006) another outbreak associated with D6–2005 (MVs/Redhill.GBR/10.06) was epidemiologically linked to Italy, although genotype D6 had not been directly detected in this country.

In addition to the main genotype D6 variants, D6–2000 and D6–2005, somewhat different viruses were found in Greece, Germany, Spain, and Luxembourg. Besides the D6–2000 virus, a second genotype D6 variant (MVs/Attica.GRC/09/06/2), with a 2-nt difference, was identified in Athens during February 2006. The same virus was also obtained from a sporadic case in Munich (Germany) 10 weeks later and caused an outbreak in the Canary Islands (Spain) after importation from Germany (Figure 2). Another genotype D6 virus identified in a sporadic case from Luxembourg in April 2006 showed a 4-nt difference from the most closely related D6 strain. In absence of an epidemiologic link, the origin of these viruses could not be determined.

### Genotype D4

**Figure 3:**
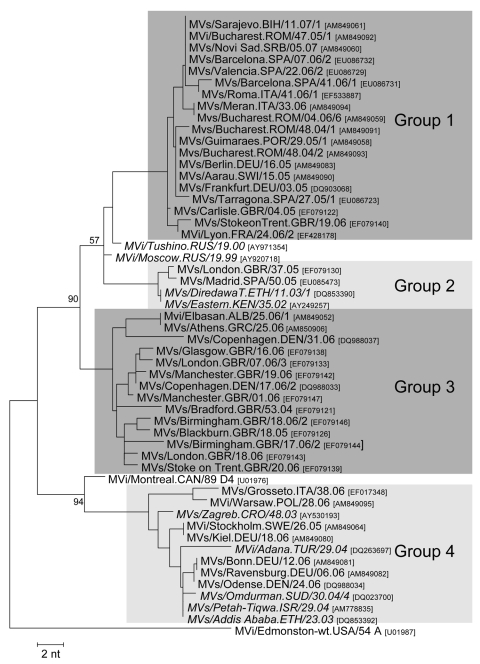
Four different genetic groups of genotype D4 identified in Europe during 2005–2006 and their closest relatives (in *italics*) identified on other continents. Tree calculation and measles virus nomenclature are as delineated in Figure 1.

In contrast to the relative genetic homogeneity of D6 viruses, at least 4 distinct genetic groups of genotype D4 were identified in Europe during 2005–2006 (Figure 3). The maximum genetic distance was 24 nt (5.3%), and the minimum distance between 2 groups was 11 nt (2.4%).

#### Group 1

A large outbreak in Romania, which included >8,000 cases and lasted from December 2004 until early 2007, started among unvaccinated members of the Roma and Sinti communities before spreading to the general population. Viruses differing by <2 nt were identified during different phases of this outbreak (Figure 3). All group 1 viruses identified in other countries differed from the most closely related Romanian virus by a maximum of 2 nt. Viruses identical to MVs/Bucharest.ROM/48.04/2 were reported from an outbreak of >220 cases in Germany (Hesse, January–April 2005; MVs/Frankfurt.DEU/03.05), which was epidemiologically linked to Romania (*18*), and from small outbreaks in Berlin (Germany), Guimares (Portugal), and an isolated case in Aarau (Switzerland) during 2005 (Figure 3). The MVs/Bucharest.ROM/47.05 variant was found in 2 different small outbreaks in Spain during weeks 7 and 22 of 2006 (MVs/Barcelona.SPA/07.06/2 and MVs/Valencia.SPA/22.06/2), the first case of which could be directly epidemiologically linked to Romania. The same variant was also found in Roma populations in Serbia as well as in Bosnia and Herzegovina during the first quarter of 2007. Another small outbreak in Cataluña (Spain) was also caused by a virus imported from Romania (MVs/Tarragona.SPA/27.05/1), with a single mutation compared with MVs/Bucharest.ROM/48.04/2. Finally, the virus that caused an outbreak of >200 cases in Barcelona (MVs/Barcelona.SPA/41.06/1; August 2006–February 2007) (*25*) was imported from Italy and had differed by 1 nt from those from an epidemic in the region of Lazio (Italy, June–November 2006), which were also taken by Roma populations to Sardinia in August 2006. A closely related genotype D4 variant, identical to MVs/Bucharest.ROM/04.06/6, caused a small outbreak during the same period in South Tyrol (Italy, Figure 3) (*26*).

The source of the Romanian viruses could not be identified. However, the homogeneity of D4 viruses identified during the Romanian outbreak, as well as the large number of measles-susceptible persons, suggests that the virus has spread within the country only after importation from another country.

#### Group 2

MV variants of a second group were found in the UK and Spain (Figure 3). MVs/Madrid.SPA/50.05 had been imported from the UK into Spain but had 1 point mutation compared with MVs/London.GBR/37.05. These viruses had 1 or 2 mutations compared with D4 viruses detected earlier in Kenya (2002) and Ethiopia (2003), which suggests an East African origin. The putative epidemiologic link to Somalia for MVs/London.GBR/37.05 also supports this hypothesis.

#### Group 3

Group 3 includes mainly viruses identified in different outbreaks and sporadic cases in the UK throughout 2005–2006, as well as in Greece, Albania, and Denmark (Figure 3). Although the source could not be identified for all group 3 viruses, the overall genetic diversity in this group (7 nt, 1.6%) suggests multiple origins. Confirmed epidemiologic links to Pakistan for MVs/London.GBR/7.06/3, MVs/Birmingham.GBR/18.06/2, MVs/Manchester.GBR/01.06, and the Danish strains suggest that most of them were imported from Central Asia, where genotype D4 is still endemic (*27*). The genotype D4 virus, which caused a small outbreak in Albania during June 2006, was imported from Greece by Roma populations.

#### Group 4

In Germany and Denmark (February and April 2006), closely related viruses of this group were found in different outbreaks and sporadic cases, which could not be epidemiologically linked to each other (Figure 3). The Danish virus (MVs/Odense.DEN/24.06) was imported from Lebanon, which is perhaps consistent with the isolation of similar D4 strains in Israel 2004 (1-nt difference). Conversely, closely related strains (with a 1- to 2-nt difference) were also seen in Ethiopia (2003) and the Sudan (2004), which suggests that the corresponding strains from Germany may also have been imported from East Africa. Another D4 variant of group 4 caused an outbreak in Tuscany (Italy, January–May 2006; MVs/Grosseto.ITA/38.06) after importation from India (*26*). This virus differed by only 3 nt from the D4 strains obtained from an outbreak in Poland that occurred from January through May 2006.

### Genotype B3

**Figure 4:**
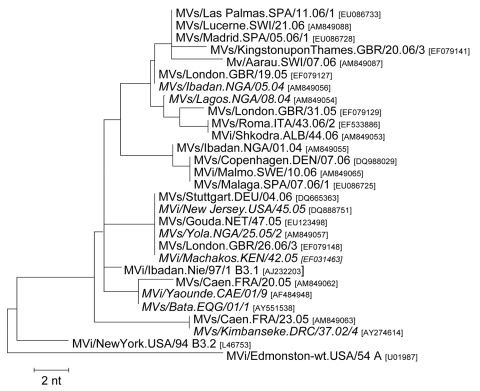
Genotype B3 variants identified in Europe during 2005–2006 and some closely related strains identified on other continents (in *italics*). Tree calculation and measles virus nomenclature are as delineated in Figure 1.

During 2005–2006, B3 strains were reported from 8 European countries, in association with outbreaks of various sizes. The maximum genetic distance between the corresponding viruses was 13 nt (2.9%) (Figure 4).

A sporadic case in the Netherlands (MVs/Gouda.NET/47.05) resulted from contact with a measles patient from Kenya at the airport in Newark, New Jersey, USA (*28*). The same virus was identified during an outbreak in Germany (January–April 2006) and the UK (June 2006) (Figure 4). Although it was not clear whether these outbreaks were due to independent importations from Africa or to MV transmission within Europe, the very high genetic diversity of MV in Africa would suggest the latter. Three other B3 variants were identified in the UK during May/June 2005 and May 2006. Although the sources are unknown, their genetic distance of 3–6 nt suggests independent importations from sub-Saharan Africa. The most closely related virus from sub-Saharan Africa (MVs/Ibadan.NGA/05.04) was identical to MVs/London.GBR/19.05, which was the cause of a major outbreak in the UK. On the other hand, 2 B3 viruses with a single mutation in comparison with MVs/London.GBR/19.05 from the UK caused 2 independent outbreaks in Spain (Madrid and Las Palmas, 2006).

Another B3 variant was detected in Denmark, Sweden, and Spain in 2006 (Figure 4). The corresponding measles cases in Denmark and Sweden were most probably epidemiologically linked (*29*), but the source of importation into Europe could not be identified. The closest non-European virus (MVs/Ibadan.NGA/01.04), which differed by 1 nt, was from Nigeria (2004).

Identical B3 strains were identified during the last quarter of 2006 in Albania and Italy (MVs/Roma.ITA/43.06/2 and MVi/Shkodra.ALB/44.06). Two considerably different B3 strains were found within a period of 3 weeks in Caen, France, in 2005. The latter clustered with viruses from either Cameroon and Equatorial Guinea or Democratic Republic of the Congo, respectively, indicating that they resulted from 2 independent introductions (Figure 4). Finally, another B3 variant was obtained from a sporadic case in Switzerland in 2006.

### Other Genotypes

In addition to D6, D4, and B3, some other genotypes (B2, D5, D8, D9, G3, H1) were found in Europe during 2005–2006 (Appendix Figure 2). Most of them were associated with sporadic cases or small outbreaks after importation of MV from other continents. The corresponding strains and confirmed sources of importation are shown in the Appendix Figure 2.

## Discussion

All 53 member states of the WHO European Region except 1 have implemented a routine vaccination program against measles, resulting in an overall regional coverage of 94% for the first dose of measles-containing vaccine. In 2006, 45% of the member states had reached an incidence of <1/1,000,000 population (Appendix Table), the threshold to declare measles elimination. To achieve regional elimination of measles by 2010, enhanced vaccination strategies are required, especially in countries with a persistently high measles incidence.

Molecular epidemiology, an essential component of measles surveillance, helps to distinguish between endemic transmission and importation of the virus. It enables identification of regions with continued MV circulation, which reflect suboptimal herd immunity. During 2005–2006, 9 of the 17 active MV genotypes (*27*) were found in the WHO European Region. The major epidemics were caused by different variants of genotypes D4, D6, and B3. The largest outbreaks occurred in the Ukraine (genotype D6, >45,000 as of June 2007), Romania (D4, >8,000 cases as of June 2007), Germany (North Rhine Westphalia, D6, ≈1,700 cases), and the Russian Federation (D6, >1,100 cases). Epidemics involving 100–500 cases were reported from the UK (B3, originated in a traveler community), Spain (B3, D4), Germany (D4, D6, B3), Italy (D4), Belarus (D6), and Greece (D6, D4). Smaller epidemics and sporadic cases were caused by either the above-mentioned genotypes or others (B2, D5, D8, D9, H1, G3).

Since the early 1990s, genotype D6 has been continuously detected in Europe (*4*–*12*). During 2005–2006, D6 strains were reported from 17 European countries and were mostly related to the large outbreak in the Ukraine (Appendix Table). The overall genetic diversity of the recent D6 strains was, however, much lower than that found during the 1990s and early 2000s, and recent D6 strains were most similar to those found during 2000–2004 mainly in the Russian Federation and Turkey (Figure 1). The low genetic diversity of D6 strains suggests that enhanced vaccination has largely reduced the cocirculation of highly diverse D6 variants in Europe. Moreover, genotypes C2 and D7, which were frequently identified in Europe until 2004 (*4*–*8*,*10*–*12*), were no longer found during the past 2 years, despite greatly improved virologic surveillance. Genotype C2 had been continuously identified in the European Region since the early 1970s, and genotype D7 had partially replaced genotypes C2 and D6 in Germany during 2000–2004 (*6*). Thus, the transmission of several indigenous MV strains from Europe has probably been interrupted by enhanced control programs.

In contrast, the prevalence of imported genotype D4 and B3 strains considerably increased during 2005–2006. Genotype D4 strains have a vast geographic distribution, and measles outbreaks associated with this genotype have been reported from all continents (*5*). D4 viruses are still endemic on the Indian subcontinent as well as in East and South Africa (*5*,*27*). Although some outbreaks and sporadic cases associated with genotype D4 had been reported from Europe, the corresponding viruses were mostly imported from other continents and differed considerably from the more recent strains (*6*,*11*,*30*). In 2005–2006, genotype D4 was found in 16 of the 27 countries in which MV has been genotyped; most of the corresponding viruses were related to the outbreak in Romania. Roma and Sinti, communities which had mostly low vaccination levels, were involved in the transmission of the Romanian D4 viruses to at least 10 other countries in Europe. The remaining genotype D4–associated cases from Europe (2005–2006) were often epidemiologically linked to the Indian Subcontinent, East Africa, and the Middle East. The high genetic diversity of the corresponding MV variants is characteristic of multiple importation events. The increased frequency of genotype D4 detection during recent years can be explained by the interruption of transmission of most of the indigenous strains, as well the introduction of genotype D4 strains into highly mobile and hard-to-reach populations with low vaccination levels.

Genotype B3 is the endemic genotype in sub-Saharan Africa; a large diversity of genotype B3 viruses continues to circulate in many countries of West and Central Africa (*31*–*37*). Before 2005, genotype B3 viruses had only been reported sporadically from Europe (*6*,*11*). The genetic diversity of the multiple B3 variants detected in Europe during 2005–2006 suggests that each one was directly or indirectly imported from sub-Saharan Africa. All of them belonged to subgroup B3.1 of genotype B3, a finding that suggests a high prevalence of B3.1 strains in Africa during 2005–2006.

Several other imported genotypes (B2, D5, D8, D9, G2, and H1), which usually do not occur in Europe, were also sporadically detected during 2005–2006. Sequence analysis confirmed or identified suspected sources of importation for most of these.

While some countries reported outbreaks caused by a single strain, other countries experienced epidemics that were caused by multiple importations of unrelated strains. As an example, the isolation of different genetic variants (D4, B3, D6–2000, D6–2005) from the 4 major epidemics in Germany (2005–2006) shows that these cases were not epidemiologically linked. The identification of multiple genotypes in Spain, Switzerland, and the UK also demonstrates multiple origins of the different outbreaks and sporadic cases. Cocirculation of different genotypes during what seemed to be 1 outbreak has been identified in Greece, Italy, and Germany. In Italy (Lazio region, 2006) and Greece (2006), viruses obtained toward the end of an epidemic (genotype B3 and D4) belonged to genotypes different from those found in the early phase (D4 and D6). In Germany very different variants of genotype D4 were found in distinct parts of the country simultaneous with the epidemic in North Rhine Westphalia. In Denmark (2006), the detection of genotypes B3 and D5 and 2 D4 variants occurred in close geographic and temporal proximity (*38*). The identification of genotype D4 in Poland did not corroborate the putative epidemiologic link between the outbreaks in Poland and the Ukraine (genotype D6) (*39*). The presence of both genotypes D4 and D6 in Poland can, however, not be excluded because no viruses were characterized from the early phase of what seemed to be 1 epidemic. The corresponding examples from Germany, Italy, Greece, Denmark, and Poland thus highlight the importance of MV genotyping during different phases of a presumably single protracted outbreak. On the other hand, MV genotyping alone may not always be sufficient to distinguish linked and unlinked outbreaks caused by multiple importations. Epidemiologic investigations in Belarus showed multiple importations from the Ukraine that caused different, unrelated outbreaks and sporadic cases in which highly similar or identical D6 variants were found.

## Conclusion

In conclusion, the absence of previously circulating genotypes (C2 and D7) and the low genetic diversity of D6 strains during 2005–2006 suggest an interruption of several MV transmission chains in the WHO European Region attributable to enhanced vaccination. However, the virus continues to be imported from other continents where measles remains highly endemic. Moreover, prolonged circulation and spread of imported strains, mostly after introduction into unvaccinated and highly mobile communities, continue to cause a high incidence of measles disease in Europe. Some countries did not report measles genotypes despite large numbers of cases (Appendix Table). As a consequence, to achieve measles elimination by 2010, measles surveillance and control need to be further optimized, and specific emphasis must be given to the vaccination of hard-to-reach populations.

## Supplementary Material

Appendix Table Numbers of measles cases, measles incidence (per 100,000 population),* and genotypes detected in the 53 member states of World Health Organization European region, 2005 and 2006.

Appendix Figure 1 Schematic view of genotype B3 (green), D4 (blue), and D6 (red) circulation in the World Health Organization European Region during 2005–2006. Bars indicate continued circulation of the same genotype in a country. The time span was delimited by the first and last case associated with highly similar variants of the same genotype and does not reflect the full duration of circulation when genotyping was not performed at the beginning and at the end of an epidemic. Dots represent single sequences obtained from sporadic cases or outbreaks. Numbers of reported measles cases per country in 2005/2006 are also shown.

Appendix Figure 2 Phylogenetic tree of all measles virus (MV) variants that were identified in Europe during 2005–2006 and that belonged to genotypes other than D4, D6, and B3. Confirmed importations from other continents are shown in brackets. Reference strains of all known MV genotypes were also included. Tree calculation and MV nomenclature are as delineated in Figure 1.
