# Supplementary material for: High Genetic Diversity of Measles Virus, World Health Organization European Region, 2005–2006
Source: Emerg Infect Dis. 2008 Jan;14(1):107–14. doi: 10.3201/eid1401.070778 (PMC2600148; doi:10.3201/eid1401.070778)
Supplement: Appendix Table — Numbers of measles cases, measles incidence (per 100,000 population),* and genotypes detected in the 53 member states of World Health Organization European region, 2005 and 2006. [file 07-0778_appT-s1.pdf]

Appendix Table. Numbers of measles cases, measles incidence (per 100,000 population),* and genotypes detected in the 53 member states of World Health Organization European region, 2005 and 2006.

	Year 2005		Year 2006		Genotypes
Country	Cases	Incidence	Cases	Incidence	
Albania	6	0.2	93	3.0	B3, D4
Andorra	0	0.0	23	34.2	–
Armenia	528	13.9	127	4.2	–
Austria	9	0.1	26	0.3	–
Azerbaijan	1200	14.5	393	4.6	D6
Belarus	1	0.0	160	1.6	D6
Belgium	26	0.3	15	0.1	–
Bosnia and Herzegovina	38	0.9	19	0.5	–
Bulgaria	3	0.0	5	0.1	D6, H1
Croatia	5	0.1	4	0.1	–
Cyprus	1	0.1	0	0.0	–
Czech Republic	2	0.0	2	0.0	–
Denmark	2	0.0	27	0.5	B3, D4, D5, D6, D9
Estonia	8	0.6	27	2.0	D6
Finland	1	0.0	0	0.0	–
France	–	–	44	0.1	D4, B3
Georgia	1351	26.4	816	18.4	–
Germany	778	1.0	2279	2.8	B2, B3, D4, D5, D6
Greece	116	1.1	512	4.6	D4, D6
Hungary	2	0.0	6	0.1	–
Iceland	0	0.0	0	0.0	–
Ireland	110	2.8	101	2.4	–
Israel	1	0.0	9	0.1	D4, D6, D8
Italy	215	0.4	249	0.4	B3, D4
Kazakhstan	15745	99.2	112	0.8	D6
Kyrgyzstan	58	1.1	26	0.5	–
Latvia	3	0.1	25	1.1	D6
Lithuania	0	0.0	113	3.3	–
Luxembourg	0	0.0	7	1.5	D6
Malta	4	1.0	0	0.0	–
Monaco	–	–	–	–	–
Montenegro	–	–	0	0.0	–
Netherlands	5	0.0	1	0.0	B3, D5
Norway	0	0.0	0	0.0	–
Poland	12	0.0	121	0.3	D4
Portugal	13	0.1	4	0.0	D4
Republic of Moldova	6	0.1	34	0.8	–
Romania	5373	24.3	2692	12.4	D4
Russian Federation	453	0.3	1147	0.8	D6, H1
San Marino	–	–	–	–	–
Serbia	0	0.0	0	0.0	–
Slovakia	0	0.0	6	0.1	–
Slovenia	0	0.0	0	0.0	–
Spain	17	0.0	349	0.8	B3, D4, D6, D8, H1
Sweden	12	0.1	19	0.2	B3, D4, D5, H1
Switzerland	62	0.9	70	1.0	B3, D4, D5, D6, D8
Tajikistan	0	0.0	19	0.3	–
The former Yugoslav Republic of Macedonia	5	0.2	1	0.0	–
Turkey	6200	8.5	34	0.0	–
Turkmenistan	0	0.0	0	0.0	–
Ukraine	2392	5.1	44534	96.8	D6
United Kingdom of Great Britain and Northern Ireland	79	0.1	773	1.3	B3, D4, D5, D6, D8, D9, G3, H1
Uzbekistan	1584	5.9	823	3.1	D6
Total European Region	36426	4.1	55847	6.3	B2, B3, D4, D5,D6, D8, D9, G3, H1

*http://data.euro.who.int/cisid.
