# Supplementary figures and images for: High Genetic Diversity of Measles Virus, World Health Organization European Region, 2005–2006
Source: Emerg Infect Dis. 2008 Jan;14(1):107–14. doi: 10.3201/eid1401.070778 (PMC2600148; doi:10.3201/eid1401.070778)

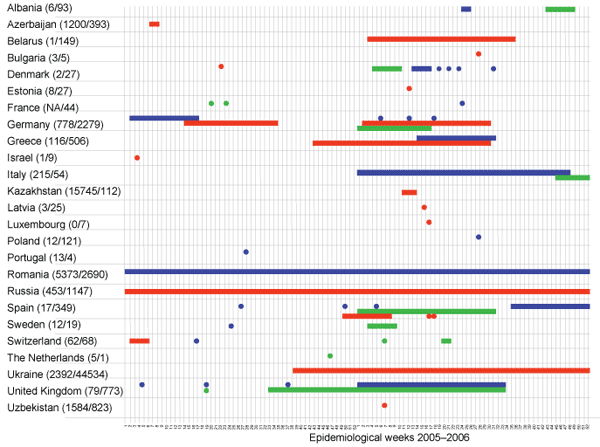

Supplement: Appendix Figure 1 — Schematic view of genotype B3 (green), D4 (blue), and D6 (red) circulation in the World Health Organization European Region during 2005–2006. Bars indicate continued circulation of the same genotype in a country. The time span was delimited by the first and last case associated with highly similar variants of the same genotype and does not reflect the full duration of circulation when genotyping was not performed at the beginning and at the end of an epidemic. Dots represent single sequences obtained from sporadic cases or outbreaks. Numbers of reported measles cases per country in 2005/2006 are also shown. [file 07-0778_app1-s2.gif]

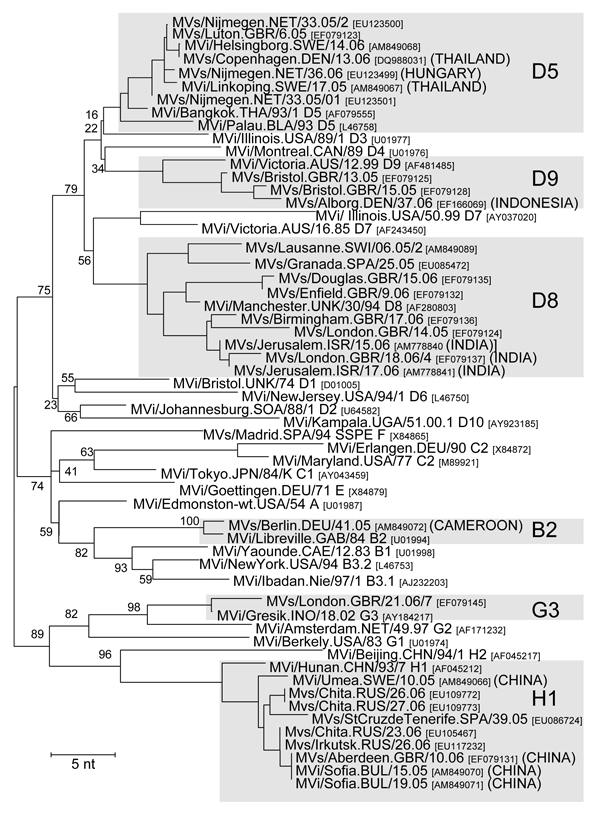

Supplement: Appendix Figure 2 — Phylogenetic tree of all measles virus (MV) variants that were identified in Europe during 2005–2006 and that belonged to genotypes other than D4, D6, and B3. Confirmed importations from other continents are shown in brackets. Reference strains of all known MV genotypes were also included. Tree calculation and MV nomenclature are as delineated in Figure 1. [file 07-0778_app2-s3.gif]
